# Beating Rate Variability of Isolated Mammal Sinoatrial Node Tissue: Insight Into Its Contribution to Heart Rate Variability

**DOI:** 10.3389/fnins.2020.614141

**Published:** 2021-02-17

**Authors:** Ori Shemla, Kenta Tsutsui, Joachim A. Behar, Yael Yaniv

**Affiliations:** ^1^Biomedical Engineering Faculty, Technion-IIT, Haifa, Israel; ^2^Department of Cardiovascular Medicine, Saitama Medical University International Medical Center, Saitama, Japan; ^3^Laboratory of Cardiovascular Science, Intramural Research Program, National Institute on Aging, Baltimore, MD, United States

**Keywords:** heart rate variability, electrogram, animal models, pacemaker, sinoatrail node

## Abstract

**Background:**

Because of the complexity of the interaction between the internal pacemaker mechanisms, cell interconnected signals, and interaction with other body systems, study of the role of individual systems must be performed under *in vivo* and *in situ* conditions. The *in situ* approach is valuable when exploring the mechanisms that govern the beating rate and rhythm of the sinoatrial node (SAN), the heart’s primary pacemaker. SAN beating rate changes on a beat-to-beat basis. However, to date, there are no standard methods and tools for beating rate variability (BRV) analysis from electrograms (EGMs) collected from different mammals, and there is no centralized public database with such recordings.

**Methods:**

We used EGM recordings obtained from control SAN tissues of rabbits (*n* = 9) and mice (*n* = 30) and from mouse SAN tissues (*n* = 6) that were exposed to drug intervention. The data were harnessed to develop a beat detector to derive the beat-to-beat interval time series from EGM recordings. We adapted BRV measures from heart rate variability and reported their range for rabbit and mouse.

**Results:**

The beat detector algorithm performed with 99% accuracy, sensitivity, and positive predictive value on the test (mouse) and validation (rabbit and mouse) sets. Differences in the frequency band cutoff were found between BRV of SAN tissue vs. heart rate variability (HRV) of *in vivo* recordings. A significant reduction in power spectrum density existed in the high frequency band, and a relative increase was seen in the low and very low frequency bands. In isolated SAN, the larger animal had a slower beating rate but with lower BRV, which contrasted the phenomena reported for *in vivo* analysis. Thus, the non-linear inverse relationship between the average HR and HRV is not maintained under *in situ* conditions. The beat detector, BRV measures, and databases were contributed to the open-source PhysioZoo software (available at: https://physiozoo.com/).

**Conclusion:**

Our approach will enable standardization and reproducibility of BRV analysis in mammals. Different trends were found between beating rate and BRV or HRV in isolated SAN tissue vs. recordings collected under *in vivo* conditions, respectively, implying a complex interaction between the SAN and the autonomic nervous system in determining HRV *in vivo*.

## Introduction

The normal heart beat dynamics involves orchestration of short- and long-scale periodic signals. These signals are generated by opening and closing of membranal channels (Adair, 2003) in heart pacemaker cells, interaction between pacemaker cells (Michaels et al., 1986), and the pacemaker cell interaction with other body systems (Yang and Xu-Friedman, 2013). To understand the role and relative contribution of each signal, experiments must be performed under *in vivo*, *in situ*, and *in vitro* conditions. When exploring the function of internal pacemaker mechanisms (see for example Yaniv et al., 2015; Behar et al., 2016), the *in vitro* conditions of isolated pacemaker cells is the optimal experimental model. However, when exploring the interconnected pacemaker cell mechanisms, the *in situ* environment of isolated sinoatrial node (SAN) tissue isolating it from all environmental effects (hormonal or nervous system) is the ideal model. While ECG recordings (i.e., *in vivo*) in a variety of mammals and electrical recordings of single pacemaker cells (*in vitro*) are routinely performed in many labs, electrical data from isolated pacemaker tissue are limited.

**FIGURE 1:**
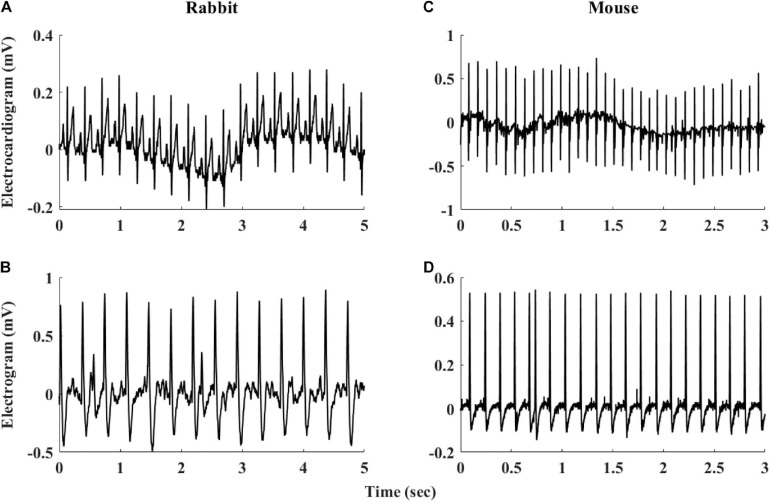
Representative examples of rabbit **(A)** ECG and **(B)** sinoatrial node EGM and mouse **(C)** ECG and **(D)** sinoatrial node EGM.

The heart rate variability (HRV, refers to variability measured under *in vivo* conditions) has been suggested as a powerful tool to explore system function (Burg et al., 1993; Bergfeldt and Haga, 2003; Rosenberg et al., 2020). HRV has been quantified *in vivo* (Goldberger et al., 2000; Behar et al., 2018b) and the beating rate variability (BRV, refers to variability measured under *in situ* or *in vitro* conditions) has been quantified in single pacemaker cells (Zaza and Lombardi, 2001; Yaniv et al., 2011). However, although the beating rate of the SAN changes on a beat-to-beat basis, BRV has not been extensively explored in isolated SAN tissue. A number of limitations hinder such research: (i) The electrogram (EGM) is used to measure electrical signals recorded on the isolated tissue surface and reflects the inner currents in this tissue. However, EGM signals differ in beat morphology and rate from *in vivo* ECG signals even if both are from the same mammal (see Figure 1). Therefore, different analysis tools are required to determine the beating rate from SAN-isolated tissue EGM than those used to determine beating rates from whole-body ECG recordings. To date, there is no database of mammalian EGM recordings available for the development of such a tool, and there are no standardized, state-of-the-art, partially or fully automated tools to analyze such recordings. (ii) Assuming that the first limitation is overcome, the beating rate of the tissue can be calculated from the EGM signals. However, there is no standard method to derive BRV from HRV, and there are no publicly available programs to analyze pacemaker tissue BRV. (iii) Isolating pacemaker tissue from healthy human patients is rare; consequently, other mammals are commonly used for cardiovascular research, with rabbits and mice being the most common mammal species used for such research. The rabbit is the smallest mammal with intracellular Ca^2+^ dynamics similar to humans (Bers, 2002; Terentyev et al., 2014; Morrissey et al., 2017). On the other hand, mouse models are commonly used for overexpression or knockout of genes implicated in human cardiovascular diseases (Thireau et al., 2008; Tzimas et al., 2017; Hook et al., 2018). Furthermore, mice are practical as aging models due to their short lifespan (Liu et al., 2014; Yaniv et al., 2016). However, tissues from different mammals differ in their beating rate, and thus, BRV parameters must be adjusted for different mammals (Behar et al., 2018b).

We aim here to overcome these three limitations and design an open-source program to analyze mammal BRV derived from pacemaker tissue EGM recordings. The new tool will be applied to (i) test the effect of drugs on BRV indices, (ii) compare the BRV indices of the different mammals, and (iii) compare BRV indices to their corresponding *in vivo* indices. This analysis will enhance our understanding of the contribution of pacemaker mechanisms to HRV *in vivo*.

## Materials and Methods

### Databases

EGM data from rabbits (*n* = 9) (Yaniv et al., 2014) and mice (*n* = 30) in basal state as well as data from mouse SAN tissues that were exposed to phosphodiesterase inhibition (using 3-isobutyl-1-methylxanthine; IBMX) (*n* = 6) were used (Yaniv et al., 2016). All animal training and validation data used in the present paper were obtained from published studies for which the respective animal protocols and experimental procedures were approved by the local research committee. Rabbit and mouse SAN were fixed in a heated bath (36 ± 0.5°C) and superfused with Tyrode’s solution (see Materials in Yaniv et al., 2014) at a rate of 4 ml/min. An insulated Teflon-coated platinum electrode with a 0.25 (rabbit)- or 0.15 (mouse)-mm diameter tip was placed at the center of the SAN to record extracellular signals using a Neurolog system NL900D (Digitimer, Hertforsdire, United Kingdom), which were recorded at 10 kHz.

### Manual Beat Detection

Because no state-of-the-art beat detector is publicly available for mammal SAN EGM data to test our suggested algorithm, beats were manually annotated. The Matlab’s “findpeaks.m” algorithm was used for initial peak detection. Then, a single trained annotator reviewed all the recordings and corrected the inaccurate annotations, i.e., false positive and false negative. These reference annotations were then used to evaluate our beat detector and to compute BRV measures. The manual annotations of the training sets were used to calculate the refractory period [minimal beating interval (BI)] and average BI of the SAN from each mammal.

### Beat Detection Algorithms

**FIGURE 2:**
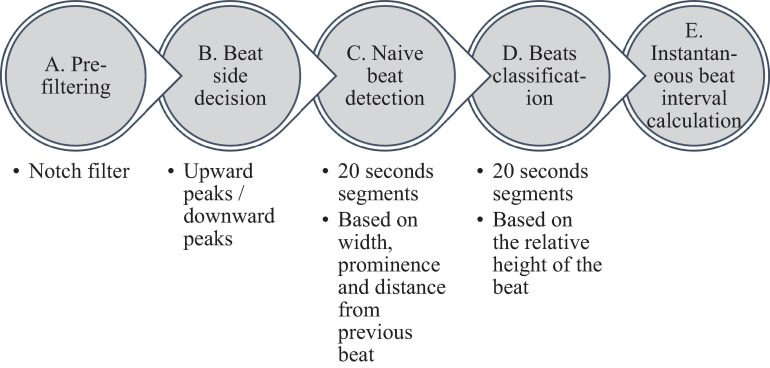
Schematic description of the beat detection algorithm.

**FIGURE 3:**
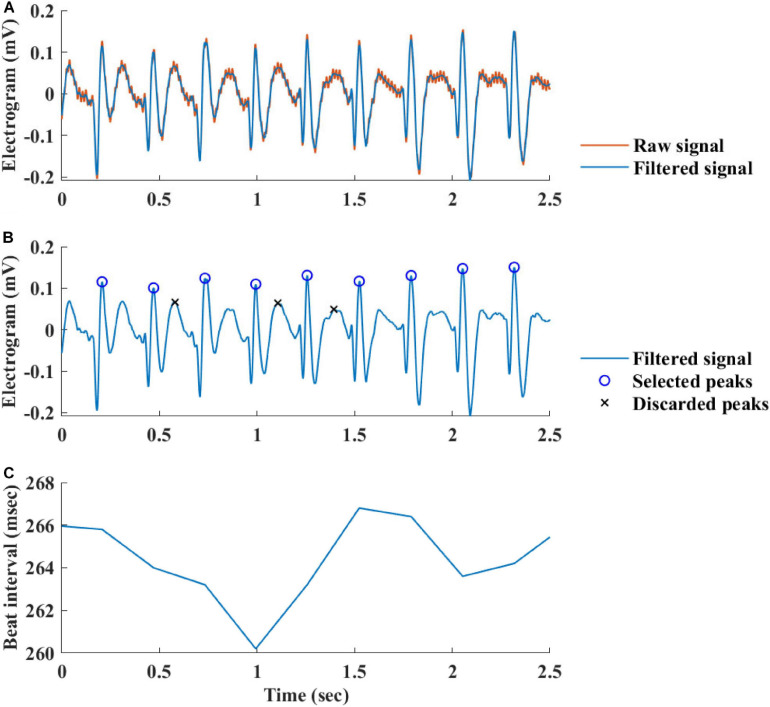
Processing steps of the EGM beat detection algorithm. **(A)** Prefiltering, **(B)** naive peak detection and classification, and **(C)** instantaneous beat interval.

Figure 2 summarizes the steps used for beat detection in the EGM record, and Figure 3 shows a representative example of the analysis step on one representative signal. In general, each EGM record went through (A) a prefiltering process to clear the data from environmental noise. A notch filter was used, which automatically identifies and reduces effects of the local electricity grid (e.g., 50/60 Hz noise) in the recording. (B) The signal upstream and downstream sign was determined based on the average frequency calculated from the power spectrum of the upward and downward parts of the filtered signal. In order to get more accurate BRV results, the side with the thinner peaks was chosen, reflected in higher average frequency. If the downward direction is preferred, the signal is reversed for the next steps. (C) Naive peak detection: Every 20 s of filtered signal was processed through Matlab’s “findpeaks.m” algorithm. The peaks were defined as any point whose distance from a prior beat is longer than the refractory period and were higher than the neighboring points, however wider than 5 data points and not wider than twice the refractory period (width measured at half the height of the peak) and more prominent than a data-derived threshold. Peak threshold = (100 - Q)^*th*^ percentile of the segment-Q^*th*^ percentile of the segment (Q = 10 for rabbit and 5 for mouse). (D) After the prominence of all of the naively annotated beats was calculated, those with peaks that were less prominent than 0.7 times the median prominence of beats were eliminated. Finally, the instantaneous BI time series was calculated.

### Beating Rate Variability Measures

#### Prefiltering

Before BRV can be calculated, two steps must be taken. First, to assure signal stationarity, a window length of 3 min for mice and 5 min for rabbits was used (Behar et al., 2018b). Second, range-based filtering was used. A certain constant range was defined, and every beat with a beating interval out of that range was excluded; for this purpose, every BI shorter than the refractory period or longer than three times the average BI of the mammal was discarded (Behar et al., 2018b). The resulting BI time series of the preprocessed signal was named NN.

#### Time Domain Measures

The majority of time domain BRV measurements is not average BI dependent, thus do not need any adjustment (see Table 1). As was pointed out before (Behar et al., 2018b), only pNNxx measures that quantify the percent of NN interval differences greater than xx milliseconds must be adjusted for different mammals. We used two approaches to define the xx: one was related to the respiratory rate *in vivo*, thus the pNNxx *in situ* was similar to the value *in vivo*, and the other was to scale the xx parameter according to the scaling ratio of the BRV AVNN relative to the reported HRV AVNN.

**TABLE 1:** Beating rate variability parameters and their derivation from HRV.

HRV measure	Units	Definition	Adjustment from *in vivo* to *in situ*
**Time domain**			
AVNN	(ms)	Average NN interval duration	No need
SDNN	(ms)	Standard deviation of NN interval duration	No need
RMSSD	(ms)	The square root of the mean of the sum of the squares of differences between adjacent NN intervals	No need
pNN_XX_	(%)	Percent of NN interval differenced greater than XX milliseconds	XX threshold, either the same or scaled according to the average BI to HR
SEM	(ms)	Standard error of the mean NN interval	No need
PIP	(%)	Percentage of inflection points in the NN interval time series	No need
IALS	(n.u.)	Inverse average length of the acceleration/deceleration segments	No need
PSS	(%)	Percentage of short segments	No need
PAS	(%)	The percentage of NN intervals in alternation segments	No need
**Frequency domain**			
Total power	(ms^2^)	Total power of the PSD function in the frequency range	No need
VLF	(ms^2^)	Power in the very low frequency band	Frequency band cutoffs
LF	(ms^2^)	Power in the low frequency band	Frequency band cutoffs
HF	(ms^2^)	Power in the high frequency band. Expected to be near 0 in BRV analysis of EGM from isolated tissue	Frequency band cutoffs
VLF norm	(%)	Power in the very low frequency band, normalized to the total power of the PSD	Frequency band cutoffs
LF norm	(%)	Power in the low frequency band, normalized to the total power in LF + HF bands	Frequency band cutoffs
HF norm	(%)	Power in the high frequency band, normalized to the total power in LF + HF bands	Frequency bands cutoffs
VLF-to-LF ratio	(n.u.)	The ratio between the power in the very low frequency and the power in the low frequency band	Frequency band cutoffs
LF-to-HF ratio	(n.u.)	The ratio between the power in the low frequency and the power in the high frequency band	Frequency band cutoffs
LF peak	(Hz)	Peak frequency in the low frequency band	Frequency band cutoffs
HF peak	(Hz)	Peak frequency in the low frequency band	Frequency bands cutoffs
**Non-linear domain**			
SD1	(ms)	NN interval standard deviation along the perpendicular to the line-of-identity in the Poincare plot	No need
SD2	(ms)	NN interval standard deviation along the line-of-identity in the Poincare plot	No need
Beta	(n.u.)	Slope of the linear interpolation of the spectrum in a log–log scale for frequencies in the VLF Beta range	VLF frequency band cutoffs
Alpha1	(n.u.)	DFA low-scale slope	DFA cutoff
Alpha2	(n.u.)	DFA high-scale slope	DFA cutoff
SampEn	(n.u.)	Sample entropy	No need

#### Frequency Domain Measures

**FIGURE 4:**
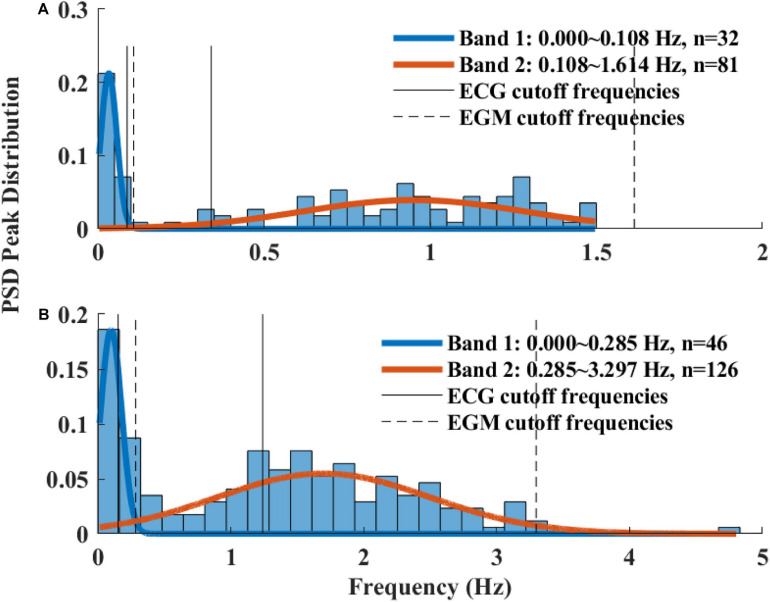
A histogram of prominent power spectrum density (PSD) peaks and a fit model of two Gaussians of **(A)** rabbit (*n* = 9) and **(B)** mouse (*n* = 12). Two frequency bands were calculated for each of the mammals (dashed lines) and compared to the bands used in ECG HRV analysis (continuous line).

The Welch’s algorithm (Welch, 1967) was used for power spectrum density (PSD) estimation. We chose this spectral estimation method over auto-regressive model (Carvalho et al., 2003; Tarvainen et al., 2006), which is a less frequently used PSD estimate, and over the Lomb method (Lomb, 1976) because of the risk of aliasing (Behar et al., 2018a). Window lengths of 3 min for mice and 5 min for rabbits were used (Behar et al., 2018b). The PSD is traditionally divided into three main bands (Malik, 1996): the very low frequency (VLF) band, the low frequency (LF) band, and the high frequency (HF) band. To determine the cutoff frequencies between the bands, we used a Gaussian mixture model (GMM) of 2 Gaussians on the histogram of all prominent peaks (see Figure 4), following the approach described in Behar et al. (2018a). To calculate the prominent peaks in each band, we used a simple peak detection algorithm to look for the 16 most prominent peaks on each of the normalized PSDs, with a threshold of 0.01. The minimal frequency was determined as one over the window length in seconds and was used to defined the lower band of VLF. The HF band was set to be between the high cutoff frequency of the LF band and 2 Hz.

#### Non-linear Domain Measures

Four measurements were used in this group: β coefficient, which corresponds to the slope of the linear interpolation of the spectrum in a log–log scale for frequencies below the upper bound of the VLF band, detrended fluctuations analysis (DFA) measures (Peng et al., 1994), Poincare analysis variation measure, and multiscale entropy (MSE) measures (Costa et al., 2005). Poincare analysis and MSE measures require no adaption. The β coefficient was estimated in the adjusted VLF band. Originally, two DFA coefficients were reported for the slopes before and after 16 beats (cutoff). We evaluated this cutoff for BRV analysis.

### User Interface

The PhysioZoo open source software (Behar et al., 2018b) was used to calculate the different BRV measures. All of our analysis tools were implemented in PhysioZoo and are open to the public.

### Performance Statistics

The quality of the beat detector was assessed by comparing between the results of the algorithm and the manually annotated beats, by using a tolerance window of 10% of the average BI of the dataset and ignoring a constant difference between the beat locations. True positive (TP) annotations, false positive (FP) annotations, and the number of missed beats [false negative (FN)] were calculated. The following statistical measurements were used to report on the quality of our beat detector:

True positive rate (TPR)—the percentage of correctly annotated beats out of all the real beats of the dataset

(1) $$T\,P\,R=100^{*}\,\frac{T\,P}{T\,P+F\,N}\%.$$

False discovery rate (FDR)—the percentage of falsely annotated beats out of all the beat annotations

(2) $$F\,D\,R=100^{*}\,\frac{F\,P}{T\,P+F\,P}\%.$$

False negative rate (FNR)—the percentage of missed beats (real and not annotated by the algorithm) out of all the real beats of the dataset

(3) $$F\,N\,R=100^{*}\,\frac{F\,N}{T\,P+F\,N}\%.$$

### General Statistics

The rank-sum test was used to define the significance level of the differences between *in situ* vs. *in vivo* conditions of the same animal and between mouse vs. rabbit SAN tissue.

## Results

This section presents the results of the performance of the beat detector on mouse and rabbit SAN tissues recordings collected under basal conditions, the range of BRV measures obtained for mouse and rabbit SAN tissues, the interpretation of BRV results in comparison to HRV, and an example of insights on pacemaker function gained from drug response BRV analysis.

### Beat Detector

To validate the ability of the beat detector to perform on unknown EGM records, we divided the mouse data into training, validation, and test datasets. The dataset was randomly divided into a training set (40%), validation set (20%), and test set (40%). The algorithm was developed using the training set and then fine-tuned by evaluating its performance on the validation set. Finally, the generalization performance of the algorithm is reported for the test set. In the case of the rabbit data, because of the limited number of animals, the data were divided into training (67%) and validation (33%) sets. Table 2 provides the performance statistics of the detector for mouse and rabbit. The beat detector algorithm very accurately detected the beat in the SAN EGM.

**TABLE 2:** Beat detector performance.

	True positive rate	False discovery rate	False negative rate
**Mouse**			
Training set (*n* = 11)	99.6 (99.0–100)%	0.5 (0.0–11.3)%	0.4 (0.0–1.0)%
Validation set (*n* = 5)	99.8 (99.5–99.9)%	0.2 (0.1–0.5)%	0.2 (0.1–0.5)%
Test set (*n* = 12)	99.4 (99.2–99.5)%	0.0 (0.0–0.0)%	0.6 (0.5–0.8)%
**Rabbit**			
Training set (*n* = 6)	99.3 (99.2–99.8)%	0.2 (0.1–0.5)%	0.7 (0.2–0.8)%
Validation set (*n* = 3)	99.1 (99.0–99.3)%	0.0 (0.0–0.2)%	0.9 (0.7–1.0)%

*All the results are presented as median and interquartile range: MED (Q1–Q3).*

### BRV Measures

**FIGURE 5:**
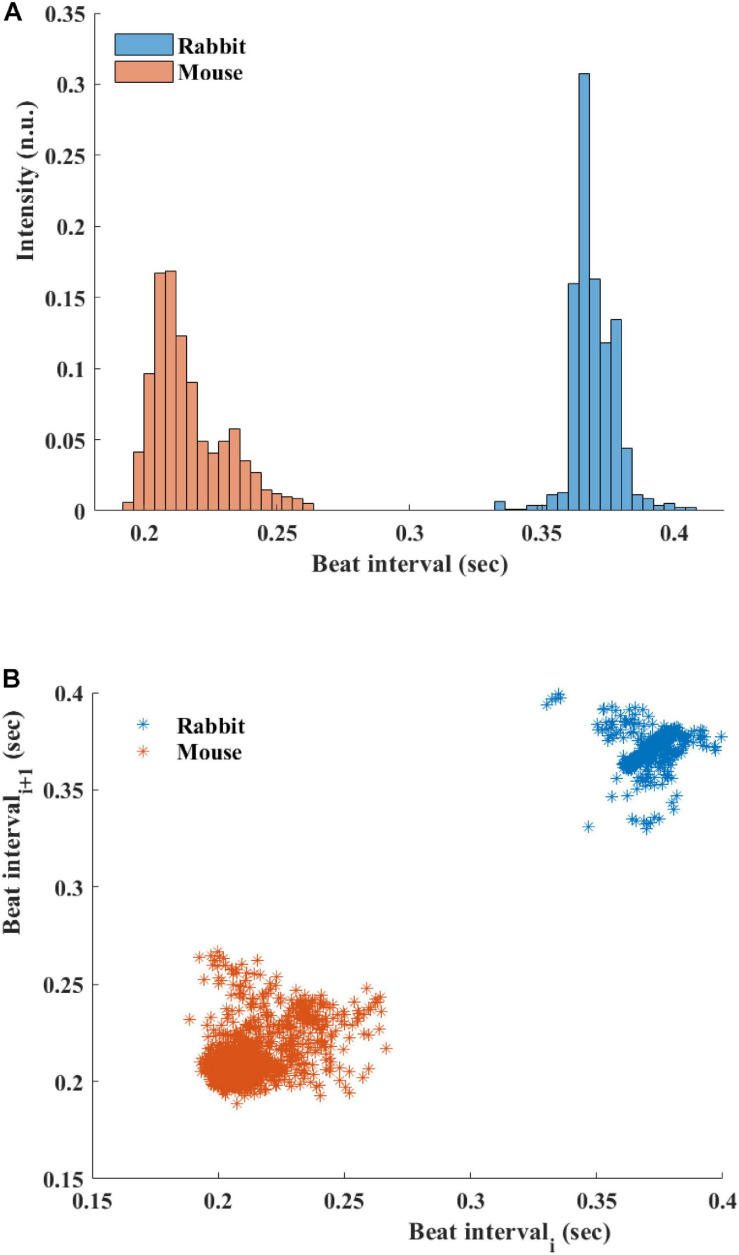
**(A)** Beating interval (BI) histogram of a representative mouse and rabbit sinoatrial node (SAN) tissue. **(B)** Poincare plots of representative examples of SAN tissue from mice and rabbits.

Figure 5A presents the BI histogram of representative examples of human-curated mouse and rabbit data. As can be seen, the BI scattering of mouse SAN tissue was higher than in the rabbit The Poincare plot (Figure 5B) was more scattered in mouse than rabbit, in accordance with the results of BRV time domain parameters.

**FIGURE 6:**
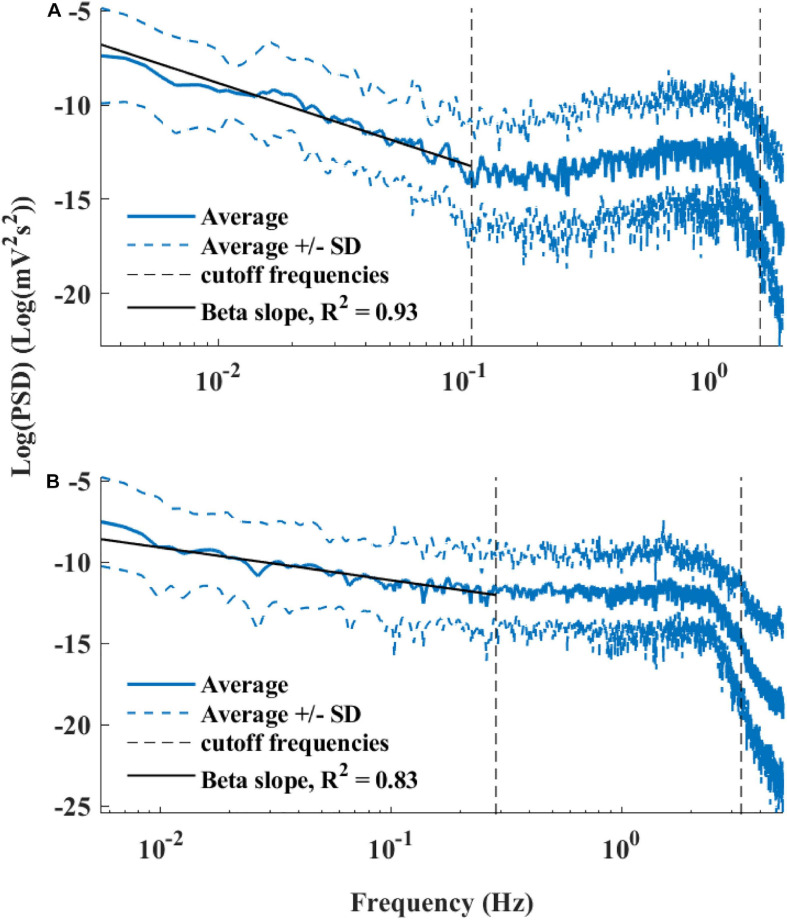
Average power spectrum density (PSD) of **(A)** rabbits (*n* = 9) and **(B)** mice (*n* = 12), in a log–log scale. The dashed lines represent the standard deviation.

**FIGURE 7:**
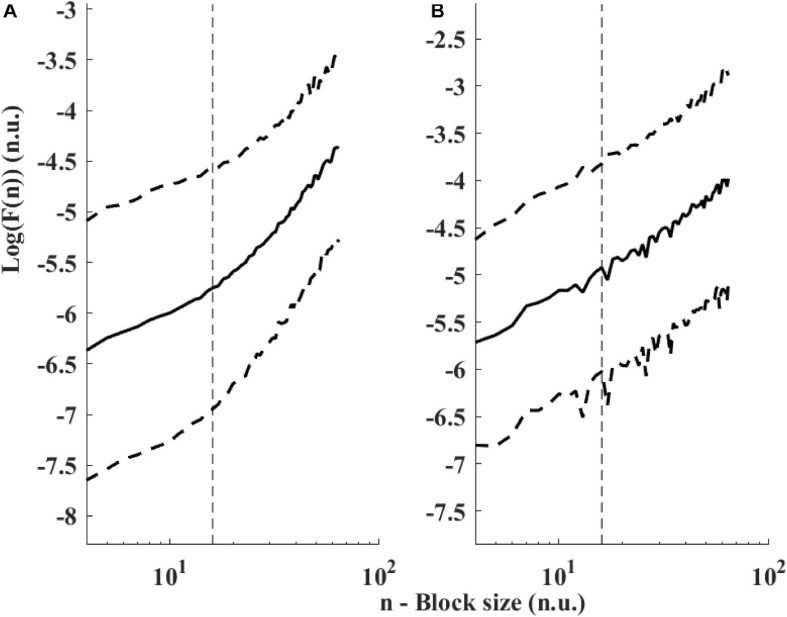
Detrended fluctuations analysis visualized. Average and standard deviation (dashed lines) of F(n) for **(A)** rabbits (*n* = 9) and **(B)** mice (*n* = 12). A change in slope is visually noticeable around 15 < *n* < 20. The traditional value used for ECG-derived HRV is *n* = 16 (Peng et al., 1995) and shown with a dashed vertical line.

Figure 6 presents the log PSD vs. log frequency for all data; a tight linear relationship between the log (PSD) and log frequency in the VLF band was achieved. Thus, we can define β as the slope of this linear regression between the log (PSD) and log frequency in the VLF band, similar to that obtained by HRV analysis. Figure 7 visualizes that the cutoff block size of DFA was similar to the cutoff under *in vivo* conditions [19.3 ± 7.6 (*n* = 7) for rabbit and 17.5 ± 9 (*n* = 11) for mouse]. Table 3 summarizes the major changes in BRV analysis under *in situ* conditions vs. *in vivo*.

**TABLE 3:** BRV analysis parameters and prefiltering parameters of *in vivo* vs. *in situ* analysis.

Parameters	Rabbit—*in vivo*	Rabbit—*in situ*	Mouse—*in vivo*	Mouse—*in situ*
VLF–LF band cutoff (Hz)	0.088	0.108	0.152	0.202
LF–HF band cutoff (Hz)	0.341	1.614	1.24	2.418
XX for pNNXX (ms)	17	24	5	12
Minimum BI to filter (s)	0.14	0.14	0.05	0.021
Maximum BI to filter (s)	0.58	0.982	0.24	0.724

*The band cutoff frequencies, the XX calculation for pNNXX, and minimum and maximum BI were calculated using the entire in situ dataset.*

**FIGURE 8:**
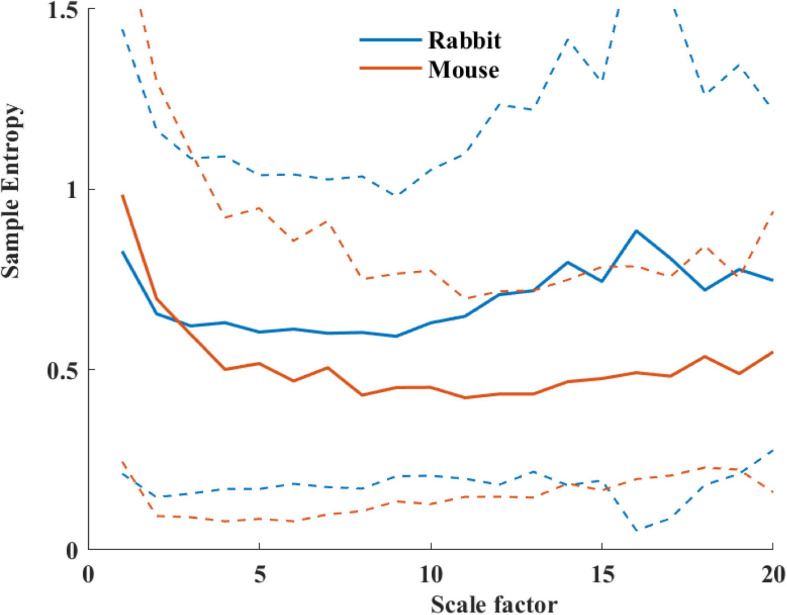
Average sample entropy as function of scale factor for mouse (*n* = 12) and rabbit (*n* = 9) SAN tissues. The dashed lines represent the standard deviation.

Table 4 summarizes the analysis results of BRV measures of rabbit and mouse. The time domain analysis found a lower variability in rabbit vs. mouse SAN tissue. In both mammals, the HF band had the least information about PSD, as expected. The PSD information was divided between the VLF and LF bands (ratio of 4:1 in mouse and ratio of 1:2 in rabbit between VLF and LF bands). The non-linear parameters showed lower complexity in mouse than rabbit. To further explore the variability and complexity observed in SAN BRV, we plotted the Poincare plot and MSE vs. order, respectively. Figure 8 shows that, in the lower scale, the MSE curve in rabbit was lower than that of mouse. However, at higher scales that represent the system complexity, the trends reverse.

**TABLE 4:** BRV measures in rabbit and mouse SAN tissues and under *in vivo* conditions.

Parameters	Rabbit *in vivo* (*p* = 33, *n* = 4)	Rabbit SAN tissue (*p* = 9, *n* = 9)	Mouse *in vivo* (*p* = 64, *n* = 8)	Mouse SAN tissue (*p* = 12, *n* = 12)
**Time domain**				
AVNN (ms)	264.95 (223.29–281.21)	335.41* (305.78–348.82)	108.50 (102.13–130.55)	187.81*,^#^ (165.47–203.14)
SDNN (ms)	10.33 (6.23–15.00)	8.69 (3.40–12.07)	10.88 (5.60–14.32)	10.43 (4.65–20.34)
RMSSD (ms)	4.64 (2.72–9.33)	4.36 (2.30–16.29)	4.73 (2.89–7.61)	9.30* (5.39–21.10)
pNN5 (%)	0.38 (0.00–1.22)	0.53 (0.00–5.89)	16.54 (3.33–29.04)	26.33^#^ (3.92–51.86)
pNN12 (%)	0.15 (0.00–0.48)	0.09 (0.00–5.06)	3.00 (0.36–8.00)	5.47 (0.32–28.89)
SEM (ms)	0.31 (0.17–0.42)	0.29 (0.11–0.38)	0.26 (0.14–0.35)	0.31 (0.14–0.67)
PIP (%)	41.68 (39.96–49.68)	62.03* (60.69–67.10)	43.09 (35.72–47.96)	68.37*,^#^ (66.47–75.05)
IALS ()	0.42 (0.40–0.50)	0.62* (0.61–0.67)	0.43 (0.36–0.48)	0.69*,^#^ (0.67–0.75)
PSS (%)	36.95 (31.19–46.56)	65.51* (62.77–75.45)	38.95 (28.48–48.67)	78.96*,^#^ (74.06–82.55)
PAS (%)	15.04 (10.30–19.59)	33.99* (32.98–35.55)	9.20 (7.07–13.24)	39.70* (35.57–56.26)
**Frequency domain**				
Total power (ms^2^)	63.39 (24.66–141.79)	23.59 (8.30–88.71)	86.62 (20.51–168.23)	99.96 (16.51–159.82)
VLF (ms^2^)	25.84 (15.75–83.72)	8.76* (2.68–20.43)	48.33 (13.50–76.58)	15.65 (2.48–77.8)
LF (ms^2^)	10.85 (5.13–25.40)	6.46 (1.42–71.72)	19.40 (5.18–56.22)	33.71 (11.39–90.98)
HF (ms^2^)	5.36 (3.36–30.86)	0.04* (0.03–1.90)	7.69 (2.13–17.76)	4.81*,^#^ (1.98–22.55)
VLF norm ()	64.21 (49.27–70.41)	65.35 (23.34–80.62)	52.51 (40.82–76.73)	14.66* (9.95–38)
LF norm ()	59.37 (49.10–74.22)	97.94* (93.84–99.18)	73.20 (62.57–80.84)	89.70*,^#^ (68.72–95.39)
HF norm ()	40.63 (25.78–50.90)	2.06* (0.82–6.16)	26.80 (19.16–37.43)	10.30*,^#^ (4.61–31.28)
VLF to LF ratio ()	2.95 (1.83–3.59)	1.93 (0.33–4.43)	1.81 (0.98–5.51)	0.25* (0.12–0.79)
LF to HF ratio ()	1.46 (0.96–2.88)	47.61* (16.51–124.13)	2.73 (1.67–4.22)	8.76*,^#^ (2.44–20.97)
LF peak (Hz)	0.12 (0.11–0.14)	0.92* (0.63–1.21)	0.25 (0.20–0.32)	1.34* (0.72–1.77)
**Non-linear domain**				
SD1 (ms)	3.28 (1.93–6.60)	3.08 (1.62–11.52)	3.34 (2.05–5.38)	6.58* (3.81–14.93)
SD2 (ms)	13.47 (8.60–18.8)	9.38 (4.41–13.15)	14.59 (7.38–19.56)	13.12 (5.36–20.49)
β ()	−0.70 (−0.82 – −0.27)	−2.23* (−2.43 – −1.26)	−1.22 (−1.68 – −0.74)	−0.64*,^#^ (−1.28 – −0.52)
Alpha1 ()	1.17 (1.04–1.30)	0.45* (0.27–0.48)	1.14 (0.97–1.25)	0.54* (0.44–0.75)
Alpha2 ()	1.00 (0.89–1.15)	1.17 (0.65–1.44)	1.03 (0.93–1.18)	0.70* (0.59–0.77)
SampEn ()	0.96 (0.60–1.18)	0.74 (0.30–1.52)	0.85 (0.54–1.29)	0.89 (0.35–1.49)

All the results are presented as median and interquartile range: MED (Q1–Q3). *p < 0.05 the in situ vs. in vivo conditions of the same mammal. ^#^p < 0.05 mouse vs. rabbit SAN tissue.

### Studying BRV in Response to Conditions That Affect Pacemaker Function

**FIGURE 9:**
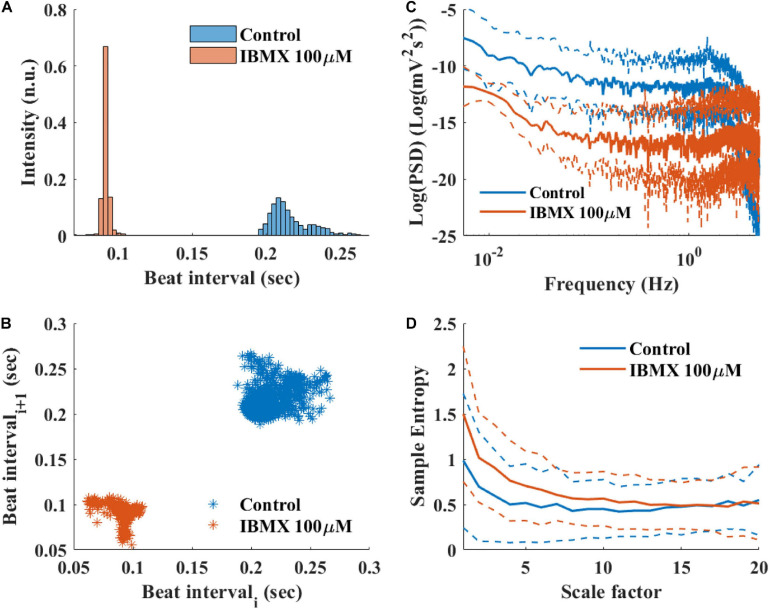
Representative BRV measures if mice SAN tissues with and without phosphodiesterase inhibition by 100 μM IBMX. Representative examples **(A)** histograms, **(B)** Poincare plots and average (control *n* = 12, drug = 6), **(C)** PSD, and **(D)** sample entropy. The dashed lines represent the standard deviation.

To study how direct changes in pacemaker function affect the BRV, phosphodiesterase activity was inhibited by applying 100 μM 3-isobutyl-1-methylxanthine (IBMX), which subsequently leads to an increase in phosphate activity and to an increase in BR. After 5 min of exposure to 100 μM IBMX, the BRV time domain measures are decreased (Table 5 and Figures 9A,B). Additionally, a reduction in the relative PSD was documented in the LF, alongside an increase in the relative PSD in the HF band in response to 100 μM IBMX. There was also a shift in the LF peak (Figure 9C). The MSE curve showed an increase in the complexity in low-scale factors in response to 100 μM IBMX and similar behavior at high scale (Figure 9D).

**TABLE 5:** BRV measures in mouse SAN tissues with and without phosphodiesterase inhibition by 100 μM IBMX.

Parameters	Mouse SAN tissue (*n* = 12)	Mouse SAN tissue + IBMX (*n* = 6)
**Time domain**		
AVNN (ms)	187.81 (165.47–203.14)	100.35 (91.42–106.69)*
SDNN (ms)	10.43 (4.65–20.34)	0.93 (0.55–4.29)*
RMSSD (ms)	9.30 (5.39–21.10)	1.48 (0.48–6.57)*
pNN5 (%)	26.33 (3.92–51.86)	0.33 (0.00–13.11)*
pNN12 (%)	5.47 (0.32–28.89)	0.00 (0.00–5.03)*
SEM (ms)	0.31 (0.14–0.67)	0.02 (0.01–0.10)*
PIP (%)	68.37 (66.47–75.05)	70.80 (64.45–72.13)
IALS ()	0.69 (0.67–0.75)	0.71 (0.65–0.72)
PSS (%)	78.96 (74.06–82.55)	80.27 (67.85–82.34)
PAS (%)	39.70 (35.57–56.26)	45.31 (38.76–47.77)
**Frequency domain**		
Total power (ms^2^)	99.96 (16.51–159.82)	0.53 (0.17–7.28)*
VLF (ms^2^)	15.65 (2.48–77.80)	0.10 (0.04–0.13)*
LF (ms^2^)	33.71 (11.39–90.98)	0.15 (0.07–1.79)*
HF (ms^2^)	4.81 (1.98–22.55)	0.31 (0.03–5.35)
VLF norm ()	14.66 (9.95–38.00)	12.64 (2.47–42.77)
LF norm ()	89.70 (68.72–95.39)	36.02 (31.85–41.91)*
HF norm ()	10.3 (4.61–31.28)	63.98 (58.09–68.15)*
VLF to LF ratio ()	0.25 (0.12–0.79)	0.46 (0.08–1.09)
LF to HF ratio ()	8.76 (2.44–20.97)	0.57 (0.47–0.72)*
LF peak (Hz)	1.34 (0.72–1.77)	2.02 (1.74–2.20)*
**Non-linear domain**		
SD1	6.58 (3.81–14.93)	1.05 (0.34–4.65)*
SD2	13.12 (5.36–20.49)	0.85 (0.58–3.88)*
Beta	−0.64 (−1.28 – −0.52)	−1.28 (−1.65 – −0.80)
Alpha1	0.54 (0.44–0.75)	0.36 (0.27–0.44)
Alpha2	0.70 (0.59–0.77)	0.45 (0.26–0.90)
SampEn	0.89 (0.35–1.49)	1.66 (1.05–2.05)

*p < 0.05.

## Discussion

### Beat Detector

We present here a novel beat detector that is suitable for EGM data. Both the beat detector and the annotated data used to evaluate its performance are available at PhysioZoo platform (Behar et al., 2018b). To the best of our knowledge, this is the first time that a beat detector and annotated database are published. The beat detector was trained on mouse data and tested on a separate database with recordings from different mice. Because the amount of rabbit data was limited, the beat detector was trained and tested on data that came from the same rabbit. Future testing with recordings from additional rabbits and validation and training with other types of mammal data (see limitation) are expected to improve our beat detector.

### BRV Measures

We present here, for the first time, a procedure for calculation of BRV measures in different mammals. The main adaptation from HRV to BRV is the changes in the band cutoff frequencies and the XX for pNNxx. Although the BR was lower under *in situ* conditions, the cutoff frequency between LF and HF was higher. These results are in contrast to the trend observed under *in vivo* conditions (Behar et al., 2018a). We also justified that the range of BI filtering should be different between *in vivo* and *in situ* conditions due to change in average BR, as expected. Under *in vivo* conditions, larger animals have a lower BR with increased HRV (Noujaim et al., 2007; Behar et al., 2018a). However, in isolated SAN tissue, a larger animal has lower BR but with lower BRV. Thus, the non-linear inverse relationship between the average HR and HRV is not maintained under *in situ* conditions. Because both the autonomic nervous system (ANS) and the SAN control the HR and HRV, lower HRV in small mammals is achieved via the balance between the sympathetic and parasympathetic systems. Thus, the ANS and the SAN have opposite effects on the HR vs. HRV relationship.

The division between the two ranges in DFA is assumed to be related to the relationship between the beating and breathing rates (Willson et al., 2002). However, we found that the cutoff between the two ranges under *in vivo* and under *in situ* conditions was similar. Thus, one may doubt that the breathing rate is the cause of the division, and it may be related to some internal pacemaker mechanism.

### BRV in Response to Conditions That Affect Pacemaker Function

We further explored the BRV in response to a drug that increases the beating rate (100 μM IBMX). As was shown before (Yaniv et al., 2016), an increase in mouse BR was associated with a decrease in the BRV time domain. Additional data regarding how drug intervention affects rabbit BRV, and BRV analysis from other mammals, will provide further insight into the BR vs. BRV relationship *in situ*.

### BRV vs. HRV Measures

Table 4 summarizes the main differences between *in vivo* and *in situ* BRV. Based on the time domain analysis, there was an increase in fragmentation parameters in recordings of isolated tissue as compared to *in vivo* conditions (Behar et al., 2018b).

Exploring the frequency domain parameters revealed interesting phenomena. The majority of information was restored in the LF and VLF bands of isolated tissue compared to *in vivo* conditions. Thus, the SAN is the main contributor to the VLF and LF bands in rabbit and to the VLF band in mice. One of the consequences of such behavior in mice and rabbits can be related to the balance between sympathetic and parasympathetic activity and to the relative magnitude of each of these activities in different mammals. The mice in our research were hosted in 20°C well below the thermoneutrality temperature and thus showed higher sympathetic activity, and sympathetic activity affects LF more than HF (Axsom et al., 2020). However, acclimation to a thermoneutral environment reversed the balance between sympathetic and parasympathetic activity (Swoap et al., 2008; Axsom et al., 2020), and thus, using SAN tissue from such mice in the future can reverse the information restored in different bands. As expected, there was almost no PSD in the HF band in both rabbit and mouse SAN tissue. Thus, the ANS is the main contributor to that band. Note that respiratory rate frequency is characteristic of the vagal activity. Because vagal activity is expressed in the HF band, it can explain the reduction in that band in the isolated tissue.

Based on the non-linear analysis parameters, the system complexity in the rabbit SAN tissue was higher as compared to *in vivo* conditions; the opposite trend was observed in mice. Thus, the system complexity is maintained mainly by the SAN in rabbit, and in mice, both SAN and ANS contribute to this complexity.

**FIGURE 10:**
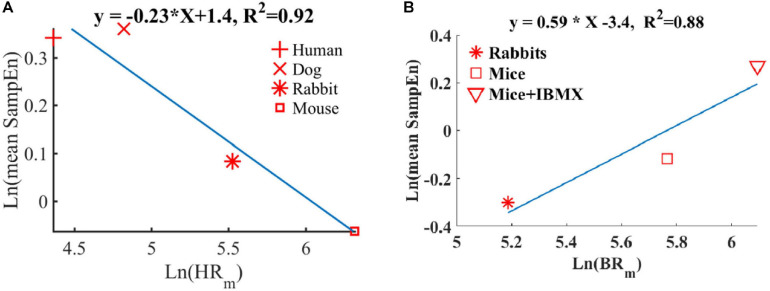
Double-logarithmic plot of the mean SampEn vs. typical **(A)** heart rate (HRm) *in vivo* (adapted from Behar et al., 2018b) and **(B)** beating rate *in vitro*.

We and others have shown before that smaller mammals have a higher HR than larger mammals (Noujaim et al., 2007; Behar et al., 2018a). We also showed that an increase in HR was associated with a decrease in sample entropy (Figure 10A). In contrast to the *in vivo* conditions, in isolated SAN tissue, the opposite relationship between entropy index and BR was observed (Figure 10B). Thus, putting the *in vivo* and SAN tissue data together on one graph is expected to show biphasic behavior for the BR and BRV. One can therefore hypothesize that when the beating rate increases *in situ*, there is an increase in short-time entropy quantified by first-order entropy. Thus, the intrinsic properties of SAN lead to an increase in entropy in response to an increase in BR. However, the fact that this relationship reverses *in vivo* implies that (i) the ANS leads to an opposite relationship between entropy and BR, and (ii) under *in vivo* conditions, the ANS controls this first-order entropy scale as was shown before (Rosenberg et al., 2020).

## Limitations

The beat detector was trained and tested on SAN from healthy animals, without any drug interventions. Future training and examination of the beat detector on EGM of SAN tissue that was exposed to a drug will be necessary to validate the use of the beat detector. Similarly, we analyzed BRV using recordings of SAN of healthy mammals. Future analysis of BRV of SAN tissue from transgenic animals or animals with cardiac diseases and comparison to the respective *in vivo* conditions will allow us to learn how changes in SAN function affects HRV *in vivo*.

## Conclusion

The approach presented here will enable standardization and reproducibility of BRV analysis in mammalian. Different trends were found between BR and BRV of isolated SAN tissue vs. HRV *in vivo* conditions, implying a complex interaction between SAN and the ANS in determining HRV *in vivo*.

## Data Availability Statement

The datasets presented in this study can be found in PhysioZoo.com.

## Ethics Statement

The animal training and validation data used in the present paper were obtained from published studies for which the respective animal protocols and experimental procedures were approved by the local research committee.

## Author Contributions

JB and YY conceived and designed the research. OS implemented the source code and interface and formatted the databases. YY drafted the manuscript. KT, JB, and YY edited and revised the manuscript. JB, YY, OS, and KT approved the final version. All authors contributed to the article and approved the submitted version.

## Conflict of Interest

The authors declare that the research was conducted in the absence of any commercial or financial relationships that could be construed as a potential conflict of interest.

## References

[B1] Adair R. K. (2003). Noise and stochastic resonance in voltage-gated ion channels. *Proc. Natl. Acad. Sci. U. S. A.* 100 12099–12104. 10.1073/pnas.2034447100 14506291 PMC218719

[B2] Axsom J. E. Nanavati A. P. Rutishauser C. A. Bonin J. E. Moen J. M. Lakatta E. G. (2020). Acclimation to a thermoneutral environment abolishes age-associated alterations in heart rate and heart rate variability in conscious, unrestrained mice. *GeroScience* 42 217–232. 10.1007/s11357-019-00126-127 31776883 PMC7031176

[B3] Behar J. Ganesan A. Zhang J. Yaniv Y. (2016). The autonomic nervous system regulates the heart rate through cAMP-PKA dependent and independent coupled-clock pacemaker cell mechanisms. *Front. Physiol.* 7:419. 10.3389/fphys.2016.00419 27729868 PMC5037226

[B4] Behar J. A. Rosenberg A. A. Shemla O. Murphy K. R. Koren G. Billman G. E. (2018a). A universal scaling relation for defining power spectral bands in mammalian heart rate variability analysis. *Front. Physiol.* 9:1001. 10.3389/fphys.2018.01001 30116198 PMC6083004

[B5] Behar J. A. Rosenberg A. A. Weiser-Bitoun I. Shemla O. Alexandrovich A. Konyukhov E. (2018b). PhysioZoo: a novel open access platform for heart rate variability analysis of mammalian electrocardiographic data. *Front. Physiol.* 9:1390. 10.3389/fphys.2018.01390 30337883 PMC6180147

[B6] Bergfeldt L. Haga Y. (2003). Power spectral and Poincare plot characteristics in sinus node dysfunction. *J. Appl. Physiol.* 94 2217–2224. 10.1152/japplphysiol.01037.2002 12576413

[B7] Bers D. M. (2002). Cardiac excitation–contraction coupling. *Nature* 415 198–205. 10.1038/415198a 11805843

[B8] Burg M. M. Jain D. Soufer R. Kerns R. D. Zaret B. L. (1993). Role of behavioral and psychological factors in mental stress-induced silent left ventricular dysfunction in coronary artery disease. *J. Am. Coll. Cardiol.* 22 440–448. 10.1016/0735-1097(93)90048-6 8335813

[B9] Carvalho J. L. A. Rocha A. F. Dos Santos I. Itiki C. Junqueira L. F. Nascimento F. A. O. (2003). “Study on the optimal order for the auto-regressive time-frequency analysis of heart rate variability,” in *Annual International Conference of the IEEE Engineering in Medicine and Biology - Proceedings*, (New York, NY: IEEE), 2621–2624. 10.1109/iembs.2003.1280453

[B10] Costa M. Goldberger A. L. Peng C.-K. (2005). Multiscale entropy analysis of biological signals. *Phys. Rev. E* 71:021906. 10.1103/PhysRevE.71.021906 15783351

[B11] Goldberger A. L. Amaral L. A. N. Glass L. Hausdorff J. M. Ivanov P. C. Mark R. G. (2000). PhysioBank, physiotoolkit, and physionet. components of a new research resource for complex physiologic signals. *Circulation* 101 215–220. 10.1161/01.CIR.101.23.e215 10851218

[B12] Hook M. Roy S. Williams E. G. Bou Sleiman M. Mozhui K. Nelson J. F. (2018). Genetic cartography of longevity in humans and mice: current landscape and horizons. *Biochim. Biophys. Acta - Mol. Basis Dis.* 1864 2718–2732. 10.1016/J.BBADIS.2018.01.026 29410319 PMC6066442

[B13] Liu J. Sirenko S. Juhaszova M. Sollott S. J. Shukla S. Yaniv Y. (2014). Age-associated abnormalities of intrinsic automaticity of sinoatrial nodal cells are linked to deficient cAMP-PKA-Ca(2+) signaling. *Am. J. Physiol. Hear. Circ. Physiol.* 306 H1385–H1397. 10.1152/ajpheart.00088.2014 24633551 PMC4024720

[B14] Lomb N. R. (1976). Least-squares frequency analysis of unequally spaced data. *Astrophys. Space Sci.* 39 447–462. 10.1007/BF00648343

[B15] Malik M. (1996). Heart rate variability. standards of measurement, physiological interpretation, and clinical use. task force of the european society of cardiology and the north american society of pacing and electrophysiology. *Eur. Heart J.* 17 354–381. 10.1111/j.1542-474X.1996.tb00275.x 8737210

[B16] Michaels D. C. Matyas E. P. Jalife J. (1986). Dynamic interactions and mutual synchronization of sinoatrial node pacemaker cells. a mathematical model. *Circ. Res.* 58 706–720. 10.1161/01.res.58.5.706 3708767

[B17] Morrissey P. J. Murphy K. R. Daley J. M. Schofield L. Turan N. N. Arunachalam K. (2017). A novel method of standardized myocardial infarction in aged rabbits. *Am. J. Physiol. Circ. Physiol.* 312 H959–H967. 10.1152/ajpheart.00582.2016 28213402 PMC5451580

[B18] Noujaim S. F. Berenfeld O. Kalifa J. Cerrone M. Nanthakumar K. Atienza F. (2007). Universal scaling law of electrical turbulence in the mammalian heart. *Proc. Natl. Acad. Sci. U S A.* 104 20985–20989. 10.1073/pnas.0709758104 18093948 PMC2409253

[B19] Peng C.-K. Buldyrev S. V. Havlin S. Simons M. Stanley H. E. Goldberger A. L. (1994). Mosaic organization of DNA nucleotides. *Phys. Rev. E* 49 1685–1689. 10.1103/PhysRevE.49.1685 9961383

[B20] Peng C.-K. Havlin S. Stanley H. E. Goldberger A. L. (1995). Quantification of scaling exponents and crossover phenomena in nonstationary heartbeat time series. *Chaos* 5 82–87. 10.1063/1.166141 11538314

[B21] Rosenberg A. A. Weiser-Bitoun I. Billman G. E. Yaniv Y. (2020). Signatures of the autonomic nervous system and the heart’s pacemaker cells in canine electrocardiograms and their applications to humans. *Sci. Rep.* 10:9971. 10.1038/s41598-020-66709-z PMC7305326 32561798

[B22] Swoap S. J. Li C. Wess J. Parsons A. D. Williams T. D. Overton J. M. (2008). Vagal tone dominates autonomic control of mouse heart rate at thermoneutrality. *Am. J. Physiol. - Hear. Circ. Physiol.* 294 1581–1588. 10.1152/ajpheart.01000.2007 18245567

[B23] Tarvainen M. P. Georgiadis S. D. Ranta-Aho P. O. Karjalainen P. A. (2006). Time-varying analysis of heart rate variability signals with a Kalman smoother algorithm. *Physiol. Meas.* 27 225–239. 10.1088/0967-3334/27/3/002 16462010

[B24] Terentyev D. Rees C. M. Li W. Cooper L. L. Jindal H. K. Peng X. (2014). Hyperphosphorylation of RyRs underlies triggered activity in transgenic rabbit model of LQT2 syndrome. *Circ. Res.* 115 919–928. 10.1161/CIRCRESAHA.115.305146 25249569 PMC4406222

[B25] Thireau J. Zhang B. L. Poisson D. Babuty D. (2008). Heart rate variability in mice: a theoretical and practical guide. *Exp. Physiol.* 93 83–94. 10.1113/expphysiol.2007.040733 17911354

[B26] Tzimas C. Johnson D. M. Santiago D. J. Vafiadaki E. Arvanitis D. A. Davos C. H. (2017). Impaired calcium homeostasis is associated with sudden cardiac death and arrhythmias in a genetic equivalent mouse model of the human HRC-Ser96Ala variant. *Cardiovasc. Res.* 113 1403–1417. 10.1093/cvr/cvx113 28859293

[B27] Welch P. D. (1967). The use of fast fourier transform for the estimation of power spectra: a method based on time averaging over short, modified periodograms. *IEEE Trans. Audio Electroacoust.* 15 70–73. 10.1109/TAU.1967.1161901

[B28] Willson K. Francis D. P. Wensel R. Coats A. J. S. Parker K. H. (2002). Relationship between detrended fluctuation analysis and spectral analysis of heart-rate variability. *Physiol. Meas.* 23 385–401. 10.1088/0967-3334/23/2/314 12051310

[B29] Yang H. Xu-Friedman M. A. (2013). Stochastic properties of neurotransmitter release expand the dynamic range of synapses. *J. Neurosci.* 33 14406–14416. 10.1523/JNEUROSCI.2487-13.2013 24005293 PMC3761050

[B30] Yaniv Y. Ahmet I. Liu J. Lyashkov A. E. Guiriba T.-R. Okamoto Y. (2014). Synchronization of sinoatrial node pacemaker cell clocks and its autonomic modulation impart complexity to heart beating intervals. *Hear. Rhythm* 11 1210–1219. 10.1016/j.hrthm.2014.03.049 24713624 PMC4065846

[B31] Yaniv Y. Ahmet I. Tsutsui K. Behar J. Moen J. M. Okamoto Y. (2016). Deterioration of autonomic neuronal receptor signaling and mechanisms intrinsic to heart pacemaker cells contribute to age-associated alterations in heart rate variability in vivo. *Aging Cell* 15 716–724. 10.1111/acel.12483 27168363 PMC4933656

[B32] Yaniv Y. Ganesan A. Yang D. Ziman B. D. Lyashkov A. E. Levchenko A. (2015). Real-time relationship between PKA biochemical signal network dynamics and increased action potential firing rate in heart pacemaker cells: kinetics of PKA activation in heart pacemaker cells. *J. Mol. Cell Cardiol.* 86 168–178. 10.1016/j.yjmcc.2015.07.024 26241846 PMC4558217

[B33] Yaniv Y. Maltsev V. A. Escobar A. L. Spurgeon H. A. Ziman B. D. Stern M. D. (2011). Beat-to-beat Ca2+-dependent regulation of sinoatrial nodal pacemaker cell rate and rhythm. *J. Mol. Cell. Cardiol.* 51 902–905. 10.1016/j.yjmcc.2011.08.029 21963899 PMC3208800

[B34] Zaza A. Lombardi F. (2001). Autonomic indexes based on the analysis of heart rate variability: a view from the sinus node. *Cardiovasc. Res.* 50 434–442. 10.1016/s0008-6363(01)00240-1 11376619

